# Demographic and socioeconomic factors associated with cognitive deficiency in patients with chronic diseases: A cross-sectional analysis from the CHARLS study

**DOI:** 10.1097/MD.0000000000049831

**Published:** 2026-07-17

**Authors:** Ru Wang, Yuxia Wang, Zhengping Tang, Qingcui Zeng

**Affiliations:** a Department of Special Medical Services, Sichuan Taikang Hospital, Chengdu, China; b Geriatric Intensive Care Unit, Sichuan Geriatric Medical Center, Sichuan Provincial People’s Hospital, University of Electronic Science and Technology of China, Chengdu, China.

**Keywords:** China Health and Retirement Longitudinal Study (CHARLS), cognitive deficiency, internal medicine diseases, risk factor, socioeconomic factor

## Abstract

This study aimed to investigate the associations between cognitive deficiency and various demographic and socioeconomic factors among individuals with chronic internal medicine diseases using data from the China Health and Retirement Longitudinal Study. A cross-sectional analysis was conducted using data from the 2020 wave of the China Health and Retirement Longitudinal Study, following the Strengthening the Reporting of Observational Studies in Epidemiology guidelines. Cognitive deficiency was assessed using a standardized composite cognitive *Z*-score, with participants scoring ≤−1.0 classified as having cognitive deficiency. To ensure robust estimates, multivariable logistic regression models were established for each sex, adjusting for standardized age and key socioeconomic covariates. A total of 4300 participants (2425 males and 1875 females) were included. Using the standardized threshold, the prevalence of cognitive deficiency was 11.4% in males and 6.9% in females. In multivariable models, nonurban residence was a significant risk factor for cognitive deficiency in both males (odds ratio [OR]: 3.37; 95% confidence interval [CI]: 2.22–5.13; *P* < .001) and females (OR: 1.93; 95% CI: 1.23–3.02; *P* = .004). Income strain was also significantly associated with cognitive deficiency in both males (OR: 1.96; 95% CI: 1.47–2.61; *P* < .001) and females (OR: 1.52; 95% CI: 1.02–2.26; *P* = .039). Marital status remained significant for males (OR: 1.43; 95% CI: 1.05–1.94; *P* = .023). Nonurban residence and financial strain are universal socioeconomic determinants of cognitive health in both male and female patients with chronic internal medicine diseases. Marital separation further increases cognitive risk in males. Targeted interventions should prioritize rural populations and economically vulnerable patients to mitigate cognitive decline.

## 1. Introduction

Cognitive function is essential for one’s daily activities and overall well-being, both socially and clinically.^[1]^ It is important for maintaining autonomy, effective communication, and decision-making, especially in healthcare settings.^[1–3]^ In clinical practice, cognitive impairment can significantly impact patient management, such as the ability to understand medical instructions, adhere to treatment plans, and provide informed consent for critical decisions, including signing advance directives, such as do-not-resuscitate orders.^[4,5]^ When viewed from a public health perspective, cognitive decline can lead to increased medical costs and a higher financial burden on the healthcare system.^[6,7]^ As populations age globally, early identification of risk factors for cognitive impairment has become a clinical and public health priority in managing such conditions and reducing adverse outcomes.^[4,7]^

While previous studies have identified multiple risk factors for cognitive impairment in the general population, such as socioeconomic status,^[8,9]^ physical activity and social engagement,^[10–12]^ and health conditions,^[9,11,13,14]^ there is a notable gap in understanding these factors specifically among individuals with chronic internal medicine conditions. The presence of chronic diseases, such as diabetes, hypertension, and cardiovascular disease, has been linked to an increased risk of cognitive decline.^[4,15,16]^ As is known, patients with chronic diseases frequently experience additional mental stress related to disease management, polypharmacy, and their diseases per se, making them a particularly vulnerable population for cognitive decline.^[4,17–20]^ Addressing this gap is crucial to developing targeted interventions and optimizing cognitive care for these patients.

To address this research gap, the present study utilizes data from the China Health and Retirement Longitudinal Study (CHARLS) to investigate the risk factors associated with cognitive deficiency in individuals with chronic internal medicine diseases. CHARLS is a nationally representative longitudinal survey that collects high-quality data on the health status, socioeconomic conditions, and cognitive function of middle-aged (45 years) and older adults in China.^[1,21,22]^ Thus, it aligns with our study objectives and supports our goal of informing interventions and protocols aimed at promoting healthy mental aging in clinical practice.

## 2. Methods

### 2.1. Study design and participants

This is a cross-sectional study utilizing data from the 2020 wave of the CHARLS survey database, reported in adherence to the Strengthening the Reporting of Observational Studies in Epidemiology guidelines. Survey participants included in the study were those diagnosed with at least 1 chronic internal medicine condition, including hypertension, dyslipidemia, diabetes or prediabetes, chronic pulmonary diseases, liver diseases, heart diseases, stroke, kidney diseases, and gastrointestinal diseases (Table S1, Supplemental Digital Content 1). Only survey respondents with complete data for all study variables were included in the analysis. Participants diagnosed with non–internal medicine conditions, such as oncologic diseases and neurological disorders, such as Alzheimer disease and Parkinson disease, were excluded.

### 2.2. Definitions of study variables

The study variables were derived from the CHARLS database and were operationally defined in an evidence-based fashion (Table S1, Supplemental Digital Content 1). The selection of these study variables, too, was evidence-based, as they had either been discussed in the literature or were clinically essential, such as basic demographics (Table S1, Supplemental Digital Content 1).

Cognitive deficiency, the outcome variable of this study, was assessed using the relevant items in the CHARLS survey questionnaire, such as immediate recall, delayed recall, orientation and attention, and visuospatial competency, following the published methods of Guo et al and Hou et al.^[22–24]^ A composite cognitive score ranging from 0 to 21 was calculated for each survey participant, with lower scores indicating greater cognitive deficiency. To preserve inherent baseline gender disparities while ensuring clinical interpretability, a pooled *Z*-score was calculated based on the mean and standard deviation of the entire cohort (N = 4300). Cognitive deficiency was defined as a standardized *Z*-score ≤ −1.0, a threshold widely used to identify mild-to-moderate cognitive deficiency in community-dwelling older adults.^[23]^ It should be noted that the cognitive threshold selected in this study represents a research definition for analytical purposes rather than a definitive clinical diagnosis.

Physical activity, referred to as “Exercise” in this study, was assessed based on the frequency of one’s exercise pattern. If a participant actively engaged in physical exercise of meaningful intensity during the past week, such as walking for the purpose of exercising, she/he was identified as physically active (Table S1, Supplemental Digital Content 1).

Contact with children was assessed by participants’ frequency of interactions with their children, either in person or virtually, such as through audio/video communication. Participants who reported neither in-person meetings nor virtual communication with their children on at least a weekly basis were categorized as “Alone,” indicating minimal contact with children. In contrast, those who reported such interaction with their children at least once per week were categorized as “Not alone,” reflecting a relatively closer parent-child connection.

Educational level was categorized into “below college” and “college and above,” wherein the degree of “Da Zhuan” – the Chinese equivalent of the associate bachelor’s degree in the West – was considered a college degree. Marital status was classified as together (married or cohabiting) or separated (widowed, divorced, or single). Smoking status was defined as a binary variable wherein only never-smokers were considered nonsmokers.

Income in this study was primarily about financial stability and stress, as it was defined based on participants’ subjective perception of their economic situation during the 2020 pandemic period. Specifically, participants were asked about their perceived ease of maintaining everyday family expenses during that time. The income level of those who reported “very easy” or “easy” was classified as “comfortable,” while that of those who reported otherwise, such as “difficult,” was classified as “tight.”

The variable of location (residence location) was defined as “City” versus “Not in City,” with the latter including both suburban and rural areas (Table S1, Supplemental Digital Content 1).

### 2.3. Study items and methodology

Since most male and female participants were married couples, the analysis was conducted separately for males and females to ensure compliance with the statistical preference that study subjects should be independent of each other.

### 2.4. Statistical analysis

To account for potential confounding from aging, age was standardized (scaled) prior to modeling. We initially explored mixed-effects logistic regression models with community and household IDs as random intercepts to account for multistage clustering; however, as the variance components were estimated as 0 (singular fit), standard multivariable logistic regression models were utilized as a more parsimonious and stable approach. Instead of univariable screening, we constructed a single, conceptually justified multivariable logistic regression model for each sex. Age was forced into the models as a core covariate. Education was excluded from the multivariable models due to complete separation (0 prevalence of cognitive deficiency in the college-educated group). Adjusted odds ratios (ORs) with 95% confidence intervals (CIs) and *P*-values were reported. Marginal effects were calculated and plotted for significant categorical predictors to visualize predicted cognitive risks. A two-sided *P*-value < .05 was considered significant. All statistical analyses were conducted using R software, version 4.4.2 (R Foundation for Statistical Computing).

## 3. Results

### 3.1. Study population and prevalence of cognitive deficiency

A total of 4300 eligible participants were included in the final analysis, comprising 2425 males (56.4%) and 1875 females (43.6%). Based on the pooled standardized cognitive *Z*-score threshold (≤ −1.0), the overall prevalence of cognitive deficiency was higher in males (11.4%, n = 277) than in females (6.9%, n = 130).

### 3.2. Baseline characteristics

Table 1 presents the baseline demographic and socioeconomic characteristics stratified by sex and cognitive status. In the male subgroup, participants with cognitive deficiency were significantly older than those with normal cognition (64.2 ± 9.4 vs 61.8 ± 8.9 years, *P* < .001). Conversely, age did not significantly differ between the cognitive deficiency and normal groups in females (58.7 ± 9.6 vs 59.7 ± 9.0 years, *P* = .251). Regarding socioeconomic factors, nonurban residence and tight income were significantly associated with higher rates of cognitive deficiency in both sexes (all *P* < .01). Specifically, 90.6% of cognitively deficient males and 80.0% of cognitively deficient females resided in nonurban areas. For males, being separated or widowed (*P* = .025) and having lower educational attainment (*P* = .045) were also significantly linked to cognitive deficiency.

**Table 1:** Baseline characteristics of male and female patients with chronic internal medicine diseases, stratified by cognitive status.*

Characteristics	Males			Females		
	Normal (N = 2148) n (%)	Cognitive deficiency (N = 277) n (%)	*P*-value	Normal (N = 1745) n (%)	Cognitive deficiency (N = 130) n (%)	*P*-value
Age (yr)	61.8 ± 8.9	64.2 ± 9.4	<.001	59.7 ± 9.0	58.7 ± 9.6	.251
Location			.001			.012
City	604 (28.1)	26 (9.4)		603 (34.6)	26 (20.0)	
Not in city	1544 (71.9)	251 (90.6)		1142 (65.4)	104 (80.0)	
Income			<.001			.003
Comfortable	914 (42.6)	75 (27.1)		749 (42.9)	39 (30.0)	
Tight	1234 (57.4)	202 (72.9)		996 (57.1)	91 (70.0)	
Marital status			.025			.707
Together	1767 (82.3)	212 (76.5)		1445 (82.8)	106 (81.5)	
Separated	381 (17.7)	65 (23.5)		300 (17.2)	24 (18.5)	
Contact with children			1			.677
Alone	1478 (68.8)	191 (69.0)		1674 (95.9)	126 (96.9)	
Not alone	670 (31.2)	86 (31.0)		71 (4.1)	4 (3.1)	
Exercise			.538			1
Active	2061 (96.0)	263 (95.0)		902 (51.7)	67 (51.5)	
Inactive	87 (4.0)	14 (5.0)		843 (48.3)	63 (48.5)	
Smoking status			.272			.092
Nonsmoker	1078 (50.2)	149 (53.8)		1640 (94.0)	117 (90.0)	
Smoker	1070 (49.8)	128 (46.2)		105 (6.0)	13 (10.0)	
Education			.045			1
College and above	33 (1.5)	0 (0.0)		7 (0.4)	0 (0.0)	
Below college	2115 (98.5)	277 (100.0)		1738 (99.6)	130 (100.0)	

Continuous variable (age) is presented as mean ± standard deviation and compared using the independent *t* test.

Categorical variables are presented as counts and percentages, n (%), and compared using the chi-square test with Monte Carlo simulation to account for small expected frequencies.

* Cognitive deficiency was defined clinically as a pooled standardized cognitive *Z*-score ≤ −1.0.

### 3.3. Multivariable logistic regression analysis

Table 2 summarizes the results of the conceptually justified multivariable logistic regression models.

**Table 2:** Multivariable logistic regression analysis of factors associated with cognitive deficiency in males and females.*

Characteristics	Adjusted odds ratio	95% CI	*P*-value
Males			
Age (per standard deviation)	1.38	1.21–1.57	<.001
Location (not in city vs city)	3.37	2.22–5.13	<.001
Income (tight vs sufficient)	1.96	1.47–2.61	<.001
Marital status (separated vs married)	1.43	1.05–1.94	.023
Contact with children (alone vs not alone)	0.89	0.67–1.18	.426
Smoking status (smoker vs nonsmoker)	0.90	0.70–1.17	.424
Exercise (inactive vs active)	1.13	0.62–2.04	.697
Females			
Age (per standard deviation)	0.89	0.73–1.09	.266
Location (not in city vs city)	1.93	1.23–3.02	.004
Income (tight vs sufficient)	1.52	1.02–2.26	.039
Marital status (separated vs married)	1.23	0.75–2.02	.406
Contact with children (alone vs not alone)	0.74	0.26–2.06	.561
Smoking status (smoker vs nonsmoker)	1.76	0.95–3.26	.071
Exercise (inactive vs active)	1	0.70–1.44	.993

Age was standardized (scaled) prior to modeling; thus, the OR for age represents the risk increase per standard deviation.

Education was excluded from the multivariable models due to complete separation (0 prevalence of cognitive deficiency in the college-educated group).

Mixed-effects models initially attempted showed 0 variance for random effects; thus, standard multivariable logistic regression was used to ensure model stability.

CI = confidence interval, OR = odds ratio.

* Cognitive deficiency was defined as a standardized cognitive *Z*-score ≤ −1.0.

**Figure 1.**
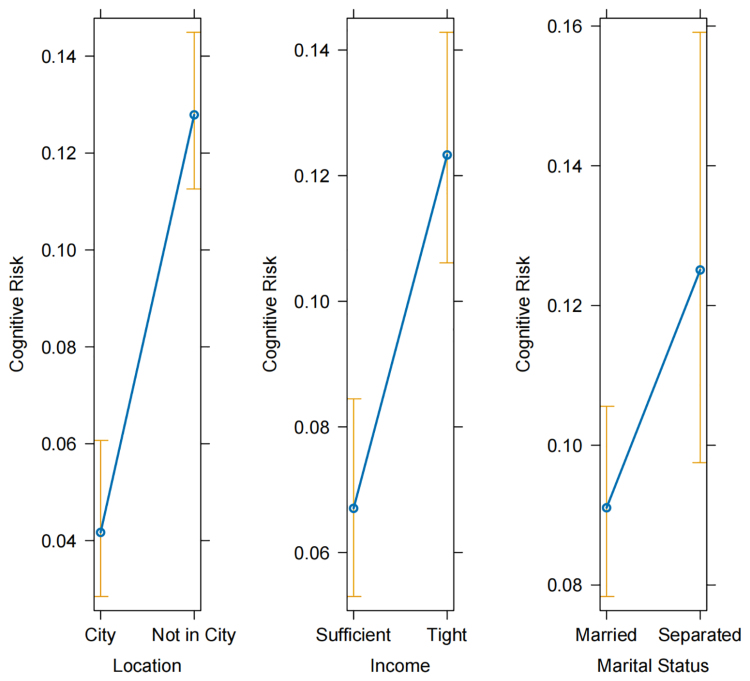
Marginal effects illustrating the predicted risk of cognitive deficiency in males based on significant socioeconomic predictors (location, income, and marital status) derived from the fully adjusted multivariable model.

In the male subgroup, after comprehensive adjustment for covariates, advanced age remained a potent predictor of cognitive deficiency (adjusted OR: 1.38, 95% CI: 1.21–1.57, *P* < .001). Nonurban residence emerged as the strongest independent risk factor, with rural males being more than 3 times as likely to exhibit cognitive deficiency as their urban counterparts (adjusted OR: 3.37, 95% CI: 2.22–5.13, *P* < .001). Additionally, tight income (adjusted OR: 1.96, 95% CI: 1.47–2.61, *P* < .001) and separated/widowed marital status (adjusted OR: 1.43, 95% CI: 1.05–1.94, *P* = .023) were independently associated with an increased risk of cognitive deficiency. Marginal effects analysis visually confirmed that the predicted probability of cognitive deficiency escalated significantly in males experiencing these combined socioeconomic disadvantages (Fig. 1).

**Figure 2.**
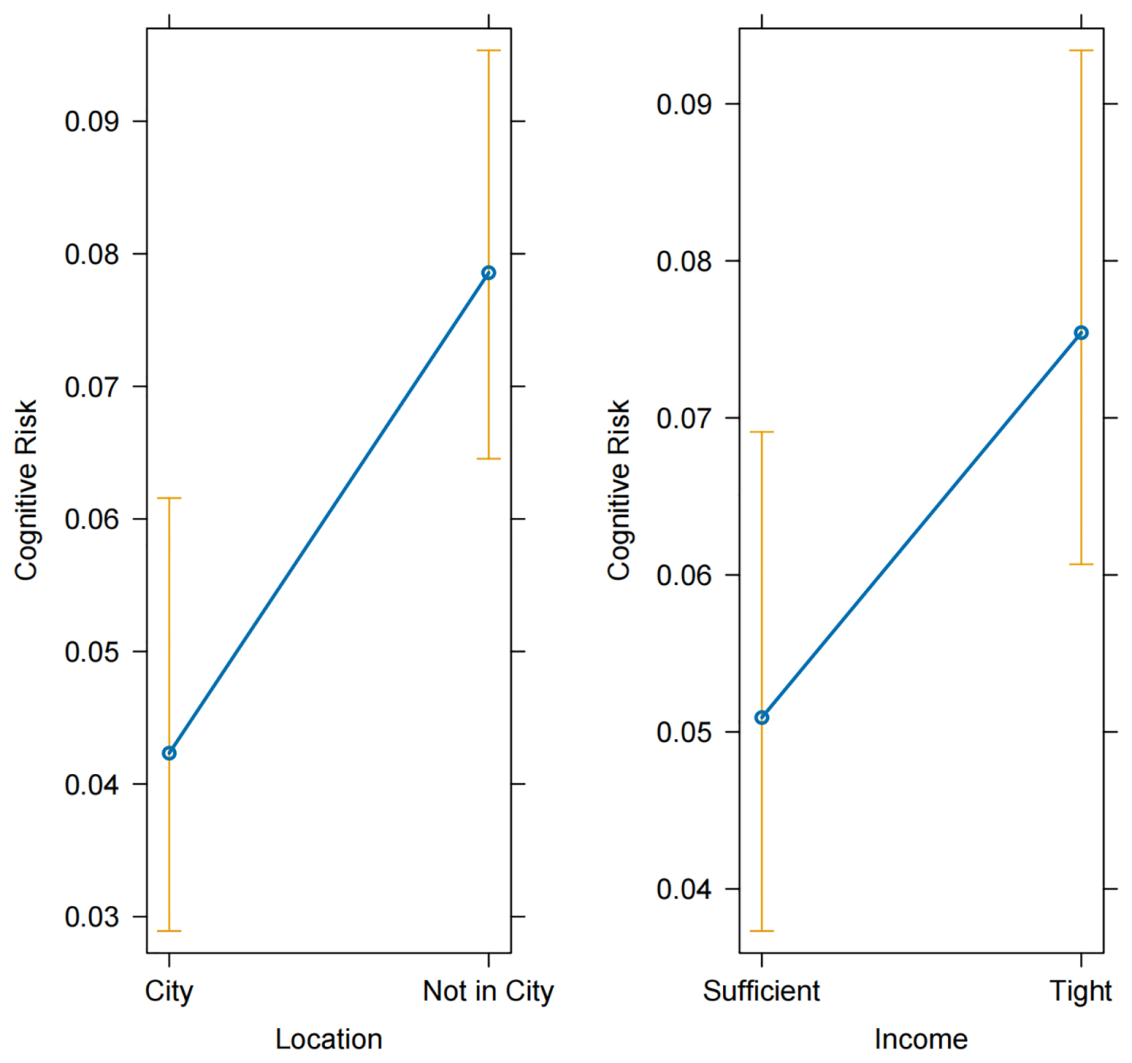
Marginal effects illustrating the predicted risk of cognitive deficiency in females based on significant socioeconomic predictors (location and income) derived from the fully adjusted multivariable model.

**Figure 3.**
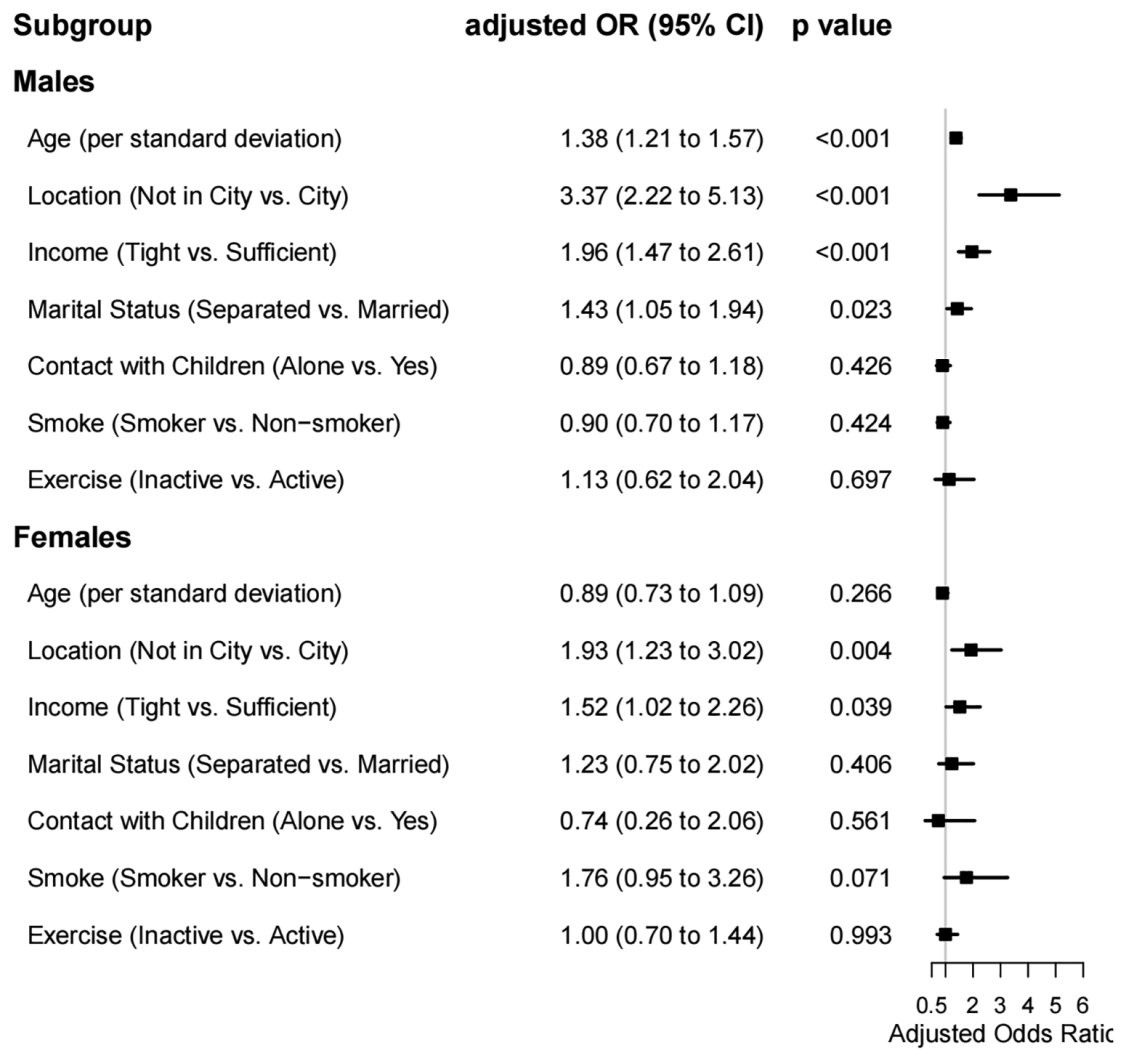
Forest plot of risk factors associated with cognitive deficiency in male and female subgroups. This forest plot visually summarizes the adjusted odds ratios (ORs) and 95% confidence intervals (CIs) derived from a single, conceptually justified multivariable logistic regression model for each sex.

In the female subgroup, socioeconomic factors demonstrated a similar but slightly attenuated pattern. Nonurban residence (adjusted OR: 1.93, 95% CI: 1.23–3.02, *P* = .004) and income strain (adjusted OR: 1.52, 95% CI: 1.02–2.26, *P* = .039) remained significant independent risk factors, driving a higher predicted cognitive risk (Figs. 2 and 3). Interestingly, unlike in males, standardized age was not a significant predictor of cognitive deficiency in females (adjusted OR: 0.89, 95% CI: 0.73–1.09, *P* = .266). Other variables, including marital status, contact with children, smoking, and exercise, did not reach statistical significance in the fully adjusted model for females. Figure 3 provides a comprehensive visual summary of these adjusted associations across both sexes based on the final models.

## 4. Discussion

The key finding of this study is the robust association between nonurban residence and cognitive vulnerability across both genders, even after adjusting for age and other socioeconomic factors. This underscores the long-term impact of geographic disparities in healthcare access and environmental stimulation. Notably, the association with marital status was attenuated in females after full adjustment, suggesting that its impact might be mediated or confounded by other life-course factors in the female cohort.

Our findings regarding the impact of residence location (in both sexes) and income level (in males) on cognitive deficiency are consistent with previous studies that have examined these socioeconomic determinants across diverse populations. Previous research has demonstrated that individuals residing in rural areas experience a higher prevalence of cognitive deficiency compared with their urban counterparts, primarily due to disparities in healthcare access, educational opportunities, and social resources.^[24,25]^ Financial strain has also been established as a critical risk factor for cognitive decline, with lower-income individuals exhibiting poorer cognitive outcomes due to restricted access to healthcare, nutritious food, and opportunities for cognitive engagement.^[26,27]^ Socioeconomic status, which encompasses both income and residence location, has been identified as a major predictor of cognitive function in studies from various countries, reinforcing the relevance of these findings across diverse cultural and economic settings.^[28,29]^ Moreover, the cumulative impact of socioeconomic adversity throughout life has been linked to accelerated cognitive decline, emphasizing the need for early interventions.^[22,28,30]^ Consistent with our findings, previous research has also found that marital status is associated with better cognitive function due to social and emotional support.^[26,27]^ Our study extends these previous results to individuals with chronic internal medicine diseases, a population that had previously received limited attention.

Several associated factors for cognitive deficiency in the general population, on the other hand, were not found to be significant in our study of individuals with chronic internal medicine diseases. For example, physical activity, which has been consistently highlighted as a protective factor against cognitive decline in the general population,^[11,31–33]^ did not exhibit a similar protective effect in our study. As another example, prior studies have consistently identified lower educational levels as a risk factor for cognitive decline. Similarly, smoking, widely recognized as a modifiable risk factor for cognitive deficiency in healthy populations,^[34]^ was not found to be significant in our study. This discrepancy may be attributed to the unique challenges faced by individuals with chronic internal medicine diseases, in whom the cumulative burden of chronic disease and complications may negate the protective benefits typically conferred by higher education or physical activity.^[35]^ These findings underscore the complexity of cognitive health in this specific population and highlight the need for tailored interventions that address their unique needs.

One merit of our study is that the study variables were defined in a clinical, rather than merely statistical, fashion. In other words, study variables were defined in a way that reflected their actual implications in clinical practice and social life. Marital status, for example, was defined in such a way that living apart was considered “separated,” but living apart due to work purposes was still considered “together,” as the latter still provides the social and emotional support that a healthy marriage is supposed to provide for one’s health, including cognitive function. It is also important to note that the definition of income level in our study aligns with the essence of income: providing for the family in a sustainable fashion. Such variable definitions are necessary for a study such as ours focusing on subjects with clinical diseases, as associations between demographic or social factors and clinical outcomes may not always be linear, such as the association between age and breast cancer survival, wherein both children and older adults have higher mortality rates than middle-aged adults.^[36]^ Our variable definitions are also in keeping with the general principle of clinical risk prediction, in which defining a variable in light of its actual clinical significance improves the validity of study results.^[36]^

From our results, there is a gender disparity in cognition-associated factors between males and females. For example, income is a significant factor in males only, while contact with children is significant only in females. This disparity aligns with the traditional gender roles and social divisions in Chinese society.

It is also worth noting that, while education is not statistically associated with cognitive deficiency in our study, no individual with a college degree has cognitive deficiency. Therefore, if the sample size were larger or the sample younger, education might become a significant protective factor against cognitive deficiency.

The implications of our study results are 2-fold. From a public health perspective, our results suggest the need to invest more resources in chronic disease patients from rural areas, which are often developing or medically underserved areas in China, and those with separated marital status or certain combinations of associated factors, such as women with minimal contact with children and men with a constrained income. In clinical practice, our results highlight the importance of recognizing the connection between these identified risk factors and patients’ cognitive competency and administering individualized care accordingly. For example, one may want to, while maintaining work efficiency, make more time for patient communication for those at risk so that negative medical consequences can be prevented.^[37]^

As a cross-sectional study based on a population survey, our study has inherent limitations. First, the reliance on self-reported data introduces the possibility of recall bias. Second, our variable operationalization – such as defining income by perceived financial strain during the 2020 pandemic period – reflects acute stressors rather than long-term structural wealth. Education categories were sparse, leading to complete separation in the models, which required the exclusion of this variable from the regression. Finally, we prioritized a standard multivariable regression approach over survey weighting to avoid severe sample attrition (nearly 40% loss due to missing weights) and selection bias, as mixed-effects testing confirmed no significant community-level clustering in this specific subpopulation. Therefore, the prevalence estimates reported in this study should not be interpreted as nationally representative.

## 5. Conclusion

Our study found that nonurban residence and financial strain are independently associated with cognitive deficiency in individuals with chronic internal medicine disorders. Public health interventions should focus on resource allocation in rural areas and financial support for vulnerable older adults to preserve cognitive health.

## Author contributions

**Conceptualization:** Ru Wang, Yuxia Wang, Zhengping Tang, Qingcui Zeng.

**Data curation:** Ru Wang, Yuxia Wang, Zhengping Tang.

**Formal analysis:** Ru Wang, Yuxia Wang.

**Investigation:** Ru Wang, Yuxia Wang, Zhengping Tang.

**Supervision:** Qingcui Zeng.



## References

[1] Wang T Wu Y Sun Y Zhai L Zhang D. A prospective study on the association between uric acid and cognitive function among middle-aged and older Chinese. J Alzheimers Dis. 2017;58:79–86. 28387669 10.3233/JAD-161243

[2] Cao H Zhao Y Chen Z. Triglyceride-glucose index predicts cognitive decline and striatal dopamine deficiency in Parkinson disease in two cohorts. NPJ Parkinsons Dis. 2025;11:240. 40804303 10.1038/s41531-025-01100-1 PMC12350777

[3] Itoh K Kuwabara A Otsuka R. Relationship between serum pyridoxal 5′-phosphate concentration and cognitive function in older Japanese. J Clin Biochem Nutr. 2025;77:79–84. 40777812 10.3164/jcbn.24-233 PMC12326249

[4] Deng Y Li N Wang Y Xiong C Zou X. Risk factors and prediction nomogram of cognitive frailty with diabetes in the elderly. Diabetes Metab Syndr Obes. 2023;16:3175–85. 37867632 10.2147/DMSO.S426315 PMC10588717

[5] LeeBKimD. Comprehensive state smoke-free policies as a predictor of cognitive impairment among older Americans: the Health and Retirement Study [published online ahead of print August 14, 2025]. Tob Control. doi: 10.1136/tc-2025-059406.10.1136/tc-2025-05940640813094

[6] Chen G Zhao M Yang K. Education exerts different effects on cognition in individuals with subjective cognitive decline and cognitive impairment: a population-based study. J Alzheimers Dis. 2021;79:653–61. 33337379 10.3233/JAD-201170

[7] Feter N de Paula D Dos Reis RCP. Racial inequities in cognitive decline of middle-aged and older adults: findings of the ELSA-Brasil study. Neurology. 2025;105:e214002. 40802924 10.1212/WNL.0000000000214002

[8] Yu L Feng J Zhou C. Cognitive function mainly shaped by socioeconomic status rather than chronic hypoxia in adolescents at high altitude. High Alt Med Biol. 2022;23:223–31. 35833789 10.1089/ham.2022.0022

[9] Larnyo E Dai B Nutakor JA Ampon-Wireko S Larnyo A Appiah R. Examining the impact of socioeconomic status, demographic characteristics, lifestyle and other risk factors on adults’ cognitive functioning in developing countries: an analysis of five selected WHO SAGE Wave 1 countries. Int J Equity Health. 2022;21:31. 35216605 10.1186/s12939-022-01622-7 PMC8876754

[10] Takeda S Fukushima H Okamoto C Kitawaki Y Nakayama S. Effects of a lifestyle development program designed to reduce the risk factors for cognitive decline on the mental health of elderly individuals. Psychogeriatrics. 2020;20:480–6. 32101630 10.1111/psyg.12538

[11] Lau H Mat Ludin AF Shahar S Badrasawi M Clark BC. Factors associated with motoric cognitive risk syndrome among low-income older adults in Malaysia. BMC Public Health. 2019;19:462. 31196017 10.1186/s12889-019-6869-z PMC6565538

[12] Caldas V Fernandes J Vafaei A. Life-space and cognitive decline in older adults in different social and economic contexts: longitudinal results from the IMIAS study. J Cross Cult Gerontol. 2020;35:237–54. 32725292 10.1007/s10823-020-09406-8

[13] Yan Z Zou X Hou X. Combined factors for predicting cognitive impairment in elderly population aged 75 years and older: from a behavioral perspective. Front Psychol. 2020;11:2217. 33013576 10.3389/fpsyg.2020.02217 PMC7511510

[14] Peng S Zhou J Xiong S. Construction and validation of cognitive frailty risk prediction model for elderly patients with multimorbidity in Chinese community based on non-traditional factors. BMC Psychiatry. 2023;23:266. 37072704 10.1186/s12888-023-04736-6 PMC10114438

[15] Heidari Z Molanouri Shamsi M Kadkhodaei A Behmanesh M Shahrbanian S Soudi S. Interplay between physical activity, inflammation, and cognitive performance in women with type 2 diabetes: an observational study focused on IL-6 pathway mediators. Diabetol Metab Syndr. 2025;17:327. 40797225 10.1186/s13098-025-01908-0 PMC12341209

[16] An W Guo D Wang J Chu X. Effects of non-pharmacological interventions on cognitive function in patients with type 2 diabetes mellitus and mild cognitive impairment: a network meta-analysis. PLoS One. 2025;20:e0329397. 40794775 10.1371/journal.pone.0329397 PMC12342316

[17] Lavielle P Talavera JO Reynoso N. Prevalence of cognitive impairment in recently diagnosed type 2 diabetes patients: are chronic inflammatory diseases responsible for cognitive decline? PLoS One. 2015;10:e0141325. 26517541 10.1371/journal.pone.0141325 PMC4627755

[18] Ihle A Ghisletta P Ballhausen N. The role of cognitive reserve accumulated in midlife for the relation between chronic diseases and cognitive decline in old age: a longitudinal follow-up across six years. Neuropsychologia. 2018;121:37–46. 30359653 10.1016/j.neuropsychologia.2018.10.013

[19] Bakouni H Gontijo Guerra S Chudzinski V Berbiche D Vasiliadis HM. One-year prospective study on the presence of chronic diseases and subsequent cognitive decline in older adults. J Public Health (Oxf). 2017;39:e170–8. 27899478 10.1093/pubmed/fdw124

[20] Lian R Liu Q Jiang G. Blood biomarkers for sarcopenia: a systematic review and meta-analysis of diagnostic test accuracy studies. Ageing Res Rev. 2024;93:102148. 38036104 10.1016/j.arr.2023.102148

[21] Zhou S Song S Jin Y Zheng ZJ. Prospective association between social engagement and cognitive impairment among middle-aged and older adults: evidence from the China Health and Retirement Longitudinal Study. BMJ Open. 2020;10:e040936. 10.1136/bmjopen-2020-040936 PMC7677353 33208332

[22] Guo S Zheng XY. New evidence of trends in cognitive function among middle-aged and older adults in China, 2011–2018: an age-period-cohort analysis. BMC Geriatr. 2023;23:498. 37605117 10.1186/s12877-023-04166-9 PMC10440902

[23] Hou DC Wei Y Sun YM Pei LJ Chen G. A cohort study of association between triglyceride glucose index-waist to height ratio and cognitive impairment in middle-aged and elderly population in China. Zhonghua Liu Xing Bing Xue Za Zhi. 2024;45:802–8. 38889979 10.3760/cma.j.cn112338-20231226-00375

[24] Yuan JL Zhao RX Ma YJ. Prevalence/potential risk factors for motoric cognitive risk and its relationship to falls in elderly Chinese people: a cross-sectional study. Eur J Neurol. 2021;28:2680–7. 33905575 10.1111/ene.14884

[25] Qi D Shi C Mao R. Sex-related associations between body height and cognitive impairment among low-income elderly adults in rural China: a population-based cross-sectional study. Biol Sex Differ. 2021;12:65. 34872609 10.1186/s13293-021-00408-w PMC8647306

[26] Zhou Y Xu Y Zhu L. The income-related distribution of cognitive function and its mobility among the Chinese elderly over a 14-year period. Psychogeriatrics. 2023;23:389–400. 36932443 10.1111/psyg.12942

[27] Steptoe A Zaninotto P. Lower socioeconomic status and the acceleration of aging: an outcome-wide analysis. Proc Natl Acad Sci U S A. 2020;117:14911–7. 32541023 10.1073/pnas.1915741117 PMC7334539

[28] Liu LY Lu Y Shen L. Prevalence, risk and protective factors for mild cognitive impairment in a population-based study of Singaporean elderly. J Psychiatr Res. 2021;145:111–7. 34894520 10.1016/j.jpsychires.2021.11.041

[29] Singh S Zhong S Rogers K Hachinski V Frisbee S. Prioritizing determinants of cognitive function in healthy middle-aged and older adults: insights from a machine learning regression approach in the Canadian Longitudinal Study on Aging. Front Public Health. 2023;11:1290064. 38186704 10.3389/fpubh.2023.1290064 PMC10768541

[30] Ni Y Zhou Y Kivimäki M. Socioeconomic inequalities in physical, psychological, and cognitive multimorbidity in middle-aged and older adults in 33 countries: a cross-sectional study. Lancet Healthy Longev. 2023;4:e618–28. 37924843 10.1016/S2666-7568(23)00195-2

[31] Boro B Saikia N. Association of multimorbidity and physical activity among older adults in India: an analysis from the Longitudinal Ageing Survey of India (2017–2018). BMJ Open. 2022;12:e053989. 10.1136/bmjopen-2021-053989 PMC9115039 35580974

[32] van Duinkerken E Ryan CM. Diabetes mellitus in the young and the old: effects on cognitive functioning across the life span. Neurobiol Dis. 2020;134:104608. 31494283 10.1016/j.nbd.2019.104608

[33] Vasiliadis HM Bélanger MF. The prospective and concurrent effect of exercise on health related quality of life in older adults over a 3 year period. Health Qual Life Outcomes. 2018;16:15. 29338743 10.1186/s12955-018-0843-9 PMC5771011

[34] Wang Q Zhou S Zhang J. Risk assessment and stratification of mild cognitive impairment among the Chinese elderly: attention to modifiable risk factors. J Epidemiol Community Health. 2023;77:521–6. 37321832 10.1136/jech-2022-219952

[35] Lian R Jiang G Liu Q. Validated tools for screening sarcopenia: a scoping review. J Am Med Dir Assoc. 2023;24:1645–54. 37567245 10.1016/j.jamda.2023.06.036

[36] Adami HO Malker B Holmberg L Persson I Stone B. The relation between survival and age at diagnosis in breast cancer. N Engl J Med. 1986;315:559–63. 3736639 10.1056/NEJM198608283150906

[37] Liu C Liu N Wang J Liu X Zhang K Li F. Hemophagocytic syndrome caused by methotrexate overdose in a total knee arthroplasty patient: a case report. JBJS Case Connect. 2020;10:e20.00068. 10.2106/JBJS.CC.20.00068 37475453

