# Supplementary material for: Demographic and socioeconomic factors associated with cognitive deficiency in patients with chronic diseases: A cross-sectional analysis from the CHARLS study
Source: Medicine (Baltimore). 2026 Jul 17;105(29):e49831. doi: 10.1097/MD.0000000000049831 (PMC13384555; doi:10.1097/MD.0000000000049831)
Supplement: Supplementary file 1 [file medi-105-e49831-s001.docx]

Table S1. Definitions of Study Variables Formulated from the CHARLS Database*

| **Study variable** | **Original variable(s) in CHARLS** | **Definition of the study variable** |
| --- | --- | --- |
| Cognitive Deficiency (the outcome variable) | Using the method of *Guo et al.* [26] and *Hou et al.* [27] whereby a composite cognitive score was calculated by incorporating the scores from episodic memory (*dc012* and *dc013*), orientation and attention (*dc001*-*dc005, dc007 series*), and visuospatial ability (*dc009*). | Cognitive deficiency: the lowest 10th percentile in composite cognitive score.  Normal: otherwise. |
| Exercise (Physical Activity) | *da033* (exercise frequency, in days per week) and *XPsyActType* (types of activities with light to vigorous intensities) | Inactive: the individual only engages in light activities, if there is any, for less than four days per week, without any moderate or vigorous activities.  Active: otherwise​. |
| Contact with Children | *ca014* (time living with children, in months per year), *ca015* and *ca016* (in-person and written/audio/video communication frequency with children, respectively, while not living together) | Alone: Not living with children and seldom communicate (not even on a weekly basis).  Not alone: living together (for at least one month/year) or having in-person/virtual communication at least once a week. |
| Income | *gf014* (The level of difficulty to cover daily expenses since the pandemic, from “very difficult” to “very easy”) | Comfortable: if “very easy” or “easy.”  Tight: otherwise. |
| Marital Status | *ba011* | Together: married, including living apart for work purpose  Separated: never married, divorced, widowed, and living apart in the sense of legal separation |
| Education Level | *ba010* | College and above: including “*Da Zhuan*,” the Chinese equivalent of associate bachelor’s degree.  Below college: otherwise. |
| Smoking Status | *da046* (smoking history), *da047* (current smoking status) | Non-smoker: never-smoker  Smoker: otherwise |
| Residence Location | *ba008* (living in urban, suburb, or rural area) | City: living in urban areas.  Not in City: otherwise. |
| *Chronic internal medicine diseases in this study were identified using the *da003* variable, excluding conditions outside the scope of general internal medicine, such as cancer, stroke, emotional and mental health problems, memory-related diseases, and Parkinson’s disease. The included conditions were hypertension, dyslipidemia, diabetes, chronic pulmonary diseases, liver diseases, heart diseases, kidney diseases, digestive system diseases, arthritis, and asthma. | | |
